# Evaluation of a Multilocus Variable-Number Tandem-Repeat Analysis Scheme for Typing *Ochrobactrum anthropi*

**DOI:** 10.3390/microorganisms12061211

**Published:** 2024-06-16

**Authors:** Yihan Wu, Liping Wang, Xiachun Hui, Guozhong Tian

**Affiliations:** 1 Inner Mongolia Autonomous Region Center for Disease Prevention and Control, Huhhot 010030, China; woyahan@163.com (Y.W.); nm-wangliping@163.com (L.W.); huichunxia@126.com (X.H.); 2 National Institute for Communicable Disease Control and Prevention, Chinese Center for Disease Control and Prevention, Beijing 102206, China

**Keywords:** Ochrobactrum anthropi, MLVA

## Abstract

*Ochrobactrum anthropi* (*O. anthropi*) is found in water, soil, plants and animals. Even though it has low virulence, it has increasingly been found to cause a number of infectious diseases in people with low immunity. The identification of *O. anthropi* mainly uses biochemical methods, such as the API 20NE or Vitek-2. The typing studies of *O. anthropi* have mainly utilized PFGE, rep-PCR, AFLP, 16s rDNA sequencing, RecA-PCR RFLP, and MALDI-TOF MS. This study aims to evaluate the polymorphisms of variable-number tandem-repeats (VNTRs) within genomic DNA of *O. anthropi* strains. The tandem repeats (TRs) in genomic DNA are discovered using Tandem Repeat Finder software (version 4.09). Twelve different VNTRs are designated and assigned to the nomenclature. The primers for PCR of 12 loci are designed. The PCR product size is converted to the number of tandem repeats in every locus. The relatedness of 65 *O. anthropi* strains from geographically different countries are analyzed by means of 12-variable-number tandem-repeat analysis(MLVA-12). A total of 51 different genotypes are found in 65 *O. anthropi* strains. These strains, which were collected from the same environmental samples, hospitals, and countries, are clustered within the same or closely genotypes. The MLVA-12 assay has a good discriminatory power for species determination, typing of *O. anthropi*, and inferring the origin of bacteria.

## 1. Introduction

*Ochrobactrum anthropi* (*O. anthropi*) is a member of the alpha-2 subdivision of *Proteobacteria*, family *Brucellaceae*. It is found in water, soil, plants and animals [1]. Even though *O. anthropi* has low virulence, it has increasingly been found to cause a number of infectious diseases in people with low immunity [2]. One hundred and seventeen cases of *O. anthropi* infections were identified and reported [3].

The availability of microbial genomic sequences has revolutionized DNA fingerprinting by facilitating the development of multilocus variable-number tandem-repeat analysis (MLVA) in recent years. On the basis of MLVA, isolates originating from restricted geographical sources can be discriminated, indicating its potential in the epidemiological tracing of bacteria transmission and in determining relationships between strains from around the world [4]. In this report, we examined tandem repeat loci in whole genomic sequences of *O. anthropi* strains in order to evaluate variable-number tandem-repeat (VNTR) markers in 65 *O. anthropi* strains.

## 2. Materials and Methods

### 2.1. Materials

PCR equipment (SensoQuest LabCycler, Goettingen, Germany), Gel imaging system (Bio-Rad ChemiDoc™ System, Hercules, CA, USA), Electrophoretic apparatus (Bio-Rad, Hercules, CA, USA). 2 *Es Taq MasterMix (Jiangsu Kangwei Century Biotechnology Co., Ltd., Taizhou, China), Primer synthesis (Bioengineering Co., Ltd. China)

### 2.2. Bacterial Strains

The whole genomic sequences of 60 *O. anthropi* strains, collected from different countries and regions around the world, were downloaded from the NCBI website (https://www.ncbi.nlm.nih.gov/, accessed on 28 April 2024). The details including the origin, year, and sources of 60 *O. anthropi* strains were downloaded. In addition, included five strains (encoded *B. anthropi* TL-1~TL-5) isolated from the same hospital and the same department (Brucellosis department) in China. *B. anthropi* TL-1 and *B. anthropi* TL-2 were isolated on 21 December 2021. *B. anthropi* TL-3, *B. anthropi* TL-4, and *B. anthropi* TL-5 were isolated on 5 January 2022. *B. anthropi* TL-1, *B. anthropi* TL-3, *B. anthropi* TL-4, *B. anthropi* TL-5 were isolated from four different patients. *B. anthropi* TL-1 was isolated from the rectal swab of a patient. *B. anthropi* TL-3, *B. anthropi* TL-4, and *B. anthropi* TL-5 were isolated from the blood of three different patients. *B. anthropi* TL-2 was isolated from discarded plastic debris from the Brucellosis department. The five strains were identifid by using Vitek-2 (BioMèrieux, Las Balmas, France). The genomic DNA of the five strains was extracted with the Promega Wizard Genomic DNA Purification kit. The five strains have not yet been subjected to whole-genome sequencing.

The genomic DNA of *Escherichia coli* ATCC 25922 strain was used as the negative control for PCR amplification in this study.

### 2.3. Identification of Tandem Repeats

The tandem repeat loci in genomic DNA were found using Tandem Repeat Finder software (version 4.09) (https://tandem.bu.edu/trf/advanced_submit, URL 3, accessed on 1 January 2024). The parameters for finding tandem repeats were as follows: the alignment parameters (match, mismatch, indels) were 2,5 and 7 respectively. The minimum alignment score to report a repeat was 30. the Maximum period size was 500. The Maximum TR array size (bp, millions) was 2. All variable-number tandem repeat loci in genomic DNA were initially screened from five genomic DNA sequences, which included chromosomes 1 and 2 (*B. anthropi* PBO, *B. anthropi* T16R-87, *B. anthropi* T210003, *B. anthropi* 1039, *B. anthropi* 1.17299). The VNTR loci were further optimized using the whole genomic sequences of 60 *O. anthropi* strains. The primers used for the amplification of all loci are described in Table 1. The principle of the designed primers was that the amplified products is suitable for both gel electrophoresis and capillary electrophoresis. The amplified products contain tandem repeats. The PCR product size can be converted to the number of tandem repeats in every locus. Each PCR reaction and gel electrophoresis at each site is independently completed.

PCR amplification was performed in a 20 μL PCR reaction tube, which contain 1ng of DNA, 10 μL of Taq mastermix, 0.4 μL (10 μ mol/L) of forward primer and reverse primer, and 8.2 μL of distilled water. The PCR reaction of each locus corresponding to each pair of primers was completed independently. An initial denaturation step was performed at 95 °C for 3 min, followed by 30 cycles of denaturation at 95 °C for 30 s, primer annealing at 55 °C for 30 s, and extension at 72 °C for 30 s, with a final extension step at 72 °C for 5 min.

### 2.4. Primer Specificity

The specificity of all primers of PCR was detected through the NCBI website (https://blast.ncbi.nlm.nih.gov/Blast, accessed on 28 April 2024).

### 2.5. Data Analysis

Clustering analyses used the categorical coefficient and UPGMA (unweighted pair group method using arithmetic averages) provided by the Bionumerics (version 4.0) software package (Applied Maths, Belgium, Brussels). Clustering analyses used the categorical coefficient and UPGMA (unweighted pair group method using arithmetic averages). The use of the categorical parameter implied that the character states were considered unordered. The same weight was given to a large or a small number of differences in the number of repeats at each locus. Maximum parsimony was performed using Bionumerics (version 4.0), running 200 bootstrap simulations and treating the data as categorical.

## 3. Results

### 3.1. Basic Information of the Strains

The countries of origin of the 65 *O. anthropi* strains include Australia (1, 1.53%), Belgium (1, 1.53%), China (13, 20%), the Czech Republic (1, 1.53%), Denmark (1, 1.53%), France (6, 9.23%), Germany (1, 1.53%), India (2, 3.08%), Norway (1, 1.53%), Pakistan (4, 6.15%), South Korea (1, 1.53%), Spain (1, 1.53%), Sweden (4, 6.15%), the UK (2, 3.08%), the USA (25, 38.46%), and Vietnam (1, 1.53%). The hosts include the environment (32, 49.23%), includinga denitrification reactor (1), metal (6),plastic debris (2), river water (1), sediments or soil (12), urban environments (8), and wood (2); Hoodstuffs (2, 3.08%), including cattle-dip trays and pasteurized milk; Human specimens (21, 32.31%), including abscesses (1), blood (8), cerebrospinal fluid (1), pleural fluid (1), rectal swabs (2), sputum (1), surface swabs (5), tracheal secretions (1), and the vagina (1); Hospital specimens (4, 6.15%), including hemodialysis apparatus (1) and washroom sinks (3).

### 3.2. Identification and Characterization of Tandem Repeat Loci

**Figure 1:**
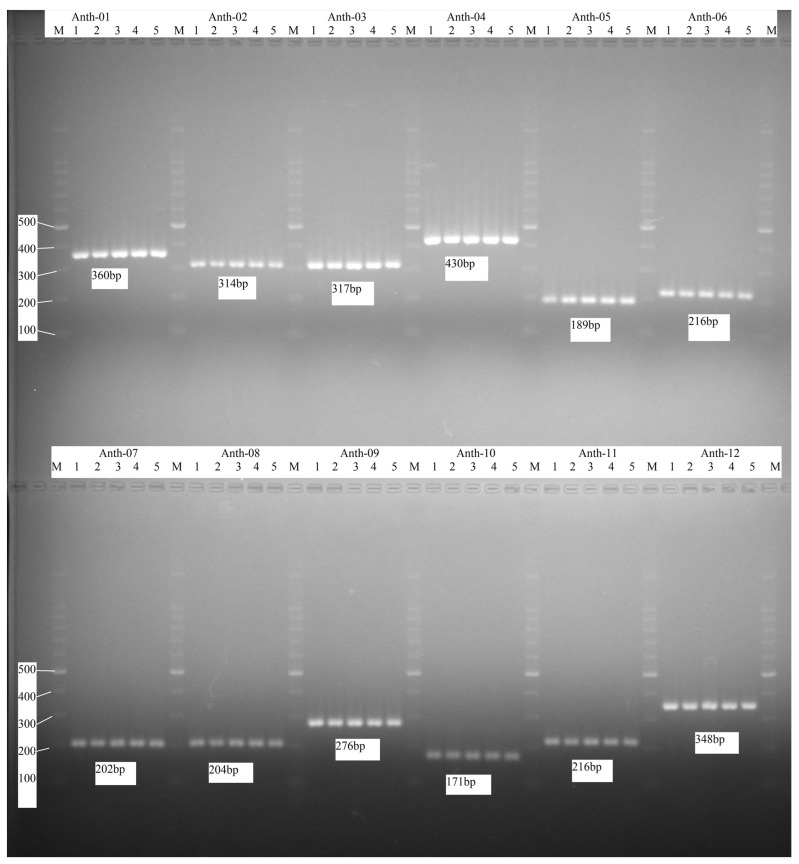
The gel electrophoresis of the PCR amplification products of 12 loci for 5 strains (*B. anthropi* TL-1~TL-5). M: DNA marker and DNA molecular weight size. The numbers 1, 2, 3, 4 and 5 correspond to *B. anthropi* TL-1, TL-2,TL-3,TL-4,and TL-5, respectively.

The base numbers and tandem repeats (TRs) of the five genomic DNA sequences (*B. anthropi* PBO, *B. anthropi* T16R-87, *B. anthropi* T210003, *B. anthropi* 1039, *B. anthropi* 1.17299) are as follows: 4,831,013 bp, 1422 TRs; 4,736,879 bp, 1355 TRs; 4,850,723 bp, 1502 TRs; 4,840,346 bp, 1465 TRs; and 4,718,862 bp, 1424 TRs, respectively. Ultimately, 12 different VNTRs were designated and assigned to the nomenclature. The set of 12 TR markers (Table 1) were used for the typing of 65 *O. anthropi* isolates. The PCR product fragment sizes were detected by using gel electrophoresis. Then, these were converted to the tandem repeat numbers according to the correspondences between the length of PCR fragments and the tandem repeat number in Table 2. The gel electrophoresis of PCR amplification products of 12 loci for 5 strains (*B. anthropi* TL-1~TL-5) is shown in Figure 1.

### 3.3. Cluster Analysis

**Figure 2:**
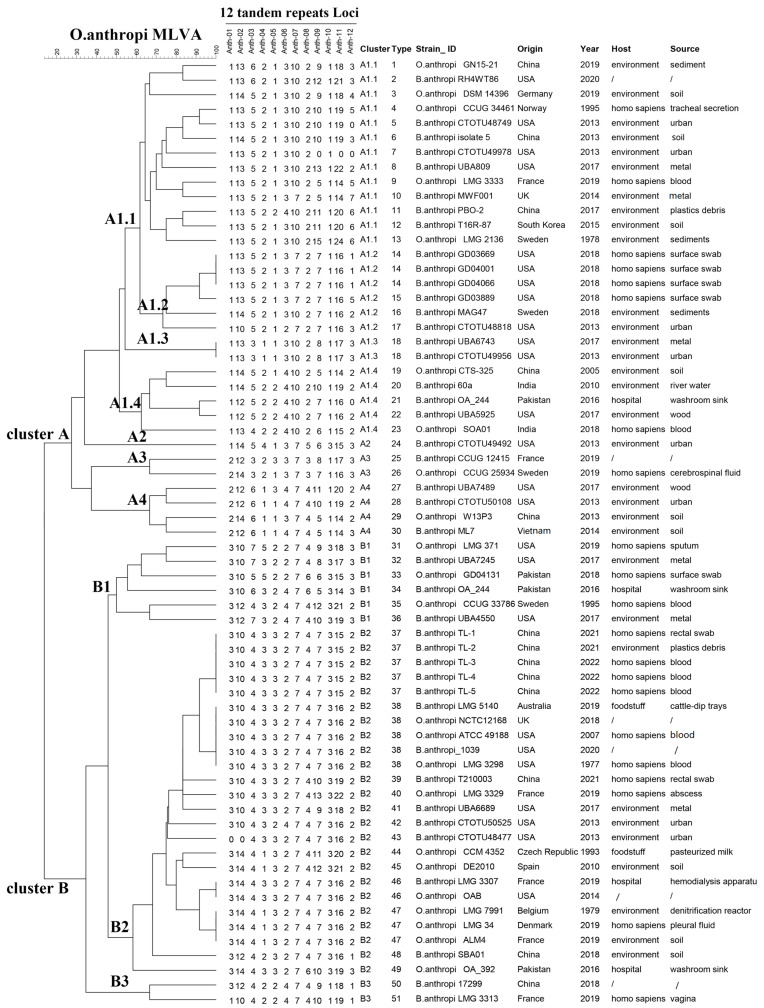
The relationshipdendrogram of 65 *O. anthropi* strains provided by the MLVA-12 assay.

The relationships between the tandem repeat numbers of 12 loci of 65 strains are determined by the construction of a neighbor-joining tree. area total of 51 different genotypes are formatted by using the MLVA-12 assay. Of these 51 genotypes, 45 genotypes are unique. Two clusters (A and B) exist for these 65 *O. anthropi* strains (Figure 2).

Cluster A is composed of 33 strains representing 30 genotypes (1–30). Cluster A is further subdivided into four major subclusters (A1–A4). Subcluster A1 can be separated into four subclusters (A1.1–1.4), composed of 26 strains. A1.1 comprises 13 strains (genotype 1–13), and 11 strains were collected from environmental samples. Two strains were from hospital patients. A1.2 is composed of six strains (genotype 14–17). Of these six strains, four strains come from surface swabs, which were taken from patients in the hospital. Among them, three strains have the same genotype (genotype 14). Only one locus (Anth-12) differs from those of the other strains. A1.3 is composed of two strains which have the same genotype (genotype 18) and were taken from environmental samples in the USA in 2013 and 2017. A1.4 comprises five strains which have different genotypes (genotype 19–23) and were taken from four countries at five different times. SubclusterA2 is composed of one strain, which was collected from environment in the USA in 2013. Subcluster A3 is composed of two strains (genotype 25–26) taken from two countries (Sweden and France)at the same time (in 2019). Subcluster A4 is composed of fourstrains which have different genotypes (genotype 27–30) and were taken from three countries at three different times.

Cluster B is composed of 32 strains representing 21 genotypes (31–51), which can be separated into three subclusters (B1–B3). Subcluster B1 comprises six strains, which have different genotypes (genotype 31–36) and were taken from three countries (Pakistan, Sweden and USA) at five different times. Two of these strains were taken from a hospital in Pakistan (33–34). Three strains were taken from sputum samples of one human patient and environmental samples in the USA in 2017 and 2019. One strain was collected from a human blood sample of in Sweden in 1995.

Subcluster B2 comprises 24 strains, representing 13 different genotypes (genotype 37–49). Of these 24 strains, 5 strains have same genotype (genotype 37), which were taken from environmental samples and four patients in the same hospital in China in this study. Genotype 38 is composed of five strains taken from three countries (Australia, the UK, and the USA). One strain was collected from cattle-dip trays. Three strains were isolated from human patients in the USA. Nine strains, collected from China, France, the USA, the Czech Republic, Pakistan, and Spain, have different genotypes (genotype 39–45,48,49). Two strains belonging to the same genotype (genotype 46) were collected from France and the USA at different times. Three strains in the same genotype (genotype 47) were collected from Belgium, Denmark, and France. Subcluster B3 is composes two strains which have two different genotypes (genotype 50–51) and were taken from two countries (China and France) at two different times.

In summary, 38.46% (25/65) of the *O. anthropi* strains were collected from hospitals. Of the 29 strains in clusters B, 55.17% (16/29) were taken from hospitals.

## 4. Discussion

The typing of the genus *Ochrobactrum* spp. Was introduced in 1998 [1]. So far, 18 species of this genus have been characterized, mainly on the basis of 16S rDNA sequencing [5], such as *O. anthropi, O. ciceri, O. cytisi, O. daejeonense, O. endophyticum, O. grignoense, O. haematophilum, O. intermedium, O. lupine, O. oryzae, O. pectoris, O. pituitosum, O. pseudintermedium, O. pseudogrignonense, O. rhizosphaerae, Ochrobactrumteleogrylli, O. thiophenivorans,* and *O. tritici*.

The biochemical identification of the genus *Ochrobactrum* spp. has been proposed, indicating that its members are non-fastidious, non-fermenting, oxidase-positive, Gram-negative rods, and resistant to beta-lactams [1,5,6]. Identification can be carried using biochemical testing kits such as API 20NE and the Vitek-2 automatic biochemical identification system [2]. API 20NE can confirm the identification at the genus level. The other biochemical reactions include urease activity, the mucoidy of the colonies, and susceptibility to colistin, bramycin and netilmicin [6]. The biochemical identification of *O. anthropi* indicates that it isGram-negative, aerobic, rod-shaped, non-pigmented, and motile, produces acid from a selection of carbohydrates, and reduces both nitrate and nitrite [6].

Molecular tools have been applied to the typing of *Ochrobactrum* spp. Early studies utilized pulsed-field gel electrophoresis, followed by PCR-RFLP and RecA-PCR RFLP (restriction fragment length polymorphism) and 16s rDNA sequencing [7,8]. The MALDI-TOF MS (matrix-assisted laser desorption/ionization–time-of-flight) assay clustered 23 *O. anthropi* strains into four distinct subgroups [9]. This technique is increasingly utilized for *Ochrobactrum* spp. identification in clinical laboratories. The multi-locus sequence analysis (MLST) approach suggests an epidemic population structure [10]. Nevertheless, there is currently no accepted scheme for phylogenetic typing to track the origin of strains. Whole-genome sequencing (WGS) and core genome MLST (cgMLST) can trace strains to their origin, but these processes can take a long time and incur a high cost [11].

Currently, the second-and third-generation sequencing results of 72 *B. anthropi* strains can be downloaded from the NCBI website. In the process of analysis, we found that two strains (*B. anthropi* SUBG007 and *B. anthropi* strain IPG1) do not have tandem repeat sequences at 12 loci. Through 16S rDNA sequencing analysis, we found that the similarity is 98% (702/716) between *B. anthropi* SUBG007 and *B.pseudogrignonense* strain ESL2. The similarity was 98% (701/716) between *B. anthropi* SUBG007 and *B.pituitosa* strain AA2. So, *B. anthropi* SUBG007 is most likely *B.pituitosa*. The similarity is 98% (1606/1641) between *B. anthropi* strain IPG1 and *B.paralicheniformis* strain NG-ABK-31. The similarity is 98% (1605/1641) between *B. anthropi* strain IPG1 and *B.intermedia* strain TSBOI. So, *B. anthropi* strain IPG1 is most likely *B.intermedia*. In addition, we could not obtain VNTRs for the other eight strains because of the incomplete sequences (some loci) of the second-generation sequencing results. So, 60 *B. anthropi* strains were analyzed usingMLVA-12. It was also proven that the MLVA-12 assay can be used to distinguish *O. anthropi* from other species of the genus *Ochrobactrum*.

This study aimed to evaluate the polymorphisms of VNTRs in *O. anthropi* isolates. MLVA-12 yielded a total of 51 genotypes with two clusters and seven subclusters. These isolates, which were collected from the same place at the same time, had same genotype, or similar genotypes, especially in hospital isolates.

The five strains (encoded *B. anthropi* TL-1~TL-5) isolated from the same hospital and the same department (Brucellosis department) in China displayed the same genotype (genotype 37). It is thus inferred that the patient infections came from this hospital and can be categorized as hospital infections. No *B. anthropi* strains were isolated from the patients and the environment in the Brucellosis department in this hospital after the thorough disinfection of the hospital.

The advantage of the MLVA typing assay is simple, operable, digitized, and enables the exchanging of data between different laboratories.

In conclusion, the MLVA-12 assay for the typing of *O. anthropi* has good discriminatory power for species determination and inferring the origin of bacteria. In the near future, it is tempting to speculate that international databases containing MLVA data will be produced.

## Figures and Tables

**Table 1:** The primers used for the amplification of tandem repeat loci.

Locus Name	ForwardPrimer	Reverse Primer	Tandem Repeat Unit	PCR Product Size (bp)	Tandem Repeat Numbers
Anth-01	5′-GAGATGTCAGCAGAAGCACTTGTG-3′	5′-AGTCGTGACTGCTCCAATCAATAGC-3′	14	360	3
Anth-02	5′-GCATATCGGCAACCGTCACCTC-3′	5′-CCATGACTATAGCTGAACGACC-3′	3	314	10
Anth-03	5′-TCGAGCCAGAGCCAGCCAT-3′	5′-ATCGTTCTCGCACTGATCGCC-3′	6	317	4
Anth-04	5′-TCTTCGCAGTGGTGATTGTCG-3′	5′-AAGGAAGTTGCAYCGGGCAG-3′	54	430	3
Anth-05	5′-CTTGAGGAYGAGCTGGAAAATC-3′	5′-ATCTGCAAGCTCGGTCTCGTC-3′	12	189	3
Anth-06	5′-GCAATGGCTCATGTTCAGAG-3′	5′-GGAAGGCAAGCGTCTGCG-3′	12	216	2
Anth-07	5′-GCCCTTGCCACCACCACC-3′	5′-CTGGAGTAATCAGAATGGTGGC-3′	3	202	7
Anth-08	5′-TTTGAGCGGACATGGCAAAG-3′	5′-CGGCTGCACTGAAGTAGTTCG-3′	13	204	4
Anth-09	5′-ATCCATGCCATCGACCTGAAAG-3′	5′-GACAAGATYGCAAGCAAGGTG-3′	6	294	10
Anth-10	5′-CTGCAAAGCGAAATATGTGGAGC-3′	5′-CGAGAATCTGCCGTTCAAGTT-3′	21	171	3
Anth-11	5′-GTCGTGGGCTTCATGATTGTGTTC-3′	5′-GACAAGATYGCAAGCAAGGTG-3′	6	240	19
Anth-12	5′-TCGCTGCCAAAAGAGAAGTGT-3′	5′-GTTATCCGTATGCTCGCCAACG-3′	125	348	2

**Table 2:** The correspondence between the length of PCR products and the number of tandem repeats in 12 loci.

Locus Name	Tandem Repeat Numbers																							
	1	2	3	4	5	6	7	8	9	10	11	12	13	14	15	16	17	18	19	20	21	22	23	24
Anth-01	332	346	360																					
Anth-02										314	317	320	323	326										
Anth-03			311	317	323	329	335																	
Anth-04	321	377	430	485	538																			
Anth-05	165	177	189																					
Anth-06		216	228	240																				
Anth-07							202			211														
Anth-08		178	191	204	217	230																		
Anth-09					264	270	276	282	288	294	300	306	312	324										
Anth-10	129		171																					
Anth-11														210	216	222	228	234	240	246	252	258	264	270
Anth-12	223	348	473	598	723	848	973																	

## Data Availability

Data are contained within the article.
